# Enhancing Performance of CdS Quantum Dot-Sensitized Solar Cells by Two-Dimensional g-C_3_N_4_ Modified TiO_2_ Nanorods

**DOI:** 10.1186/s11671-016-1677-1

**Published:** 2016-10-18

**Authors:** Qiqian Gao, Shihan Sun, Xuesong Li, Xueyu Zhang, Lianfeng Duan, Wei Lü

**Affiliations:** Key Laboratory of Advanced Structural Materials, Ministry of Education, Changchun University of Technology, Changchun, 130012 China

**Keywords:** g-C_3_N_4_, TiO_2_ nanorod arrays, Photoelectronical performance, Solar cells

## Abstract

In present work, two-dimensional g-C_3_N_4_ was used to modify TiO_2_ nanorod array photoanodes for CdS quantum dot-sensitized solar cells (QDSSCs), and the improved cell performances were reported. Single crystal TiO_2_ nanorods are prepared by hydrothermal method on transparent conductive glass and spin-coated with g-C_3_N_4_. CdS quantum dots were deposited on the g-C_3_N_4_ modified TiO_2_ photoanodes via successive ionic layer adsorption and reaction method. Compared with pure TiO_2_ nanorod array photoanodes, the g-C_3_N_4_ modified photoanodes showed an obvious improvement in cell performances, and a champion efficiency of 2.31 % with open circuit voltage of 0.66 V, short circuit current density of 7.13 mA/cm^2^, and fill factor (FF) of 0.49 was achieved, giving 23 % enhancement in cell efficiency. The improved performances were due to the matching conduction bands and valence bands of g-C_3_N_4_ and TiO_2_, which greatly enhanced the separation and transfer of the photogenerated electrons and holes and effectively suppressed interfacial recombination. Present work provides a new direction for improving performance of QDSSCs.

## Background

As one kind of novel solar cells, quantum dot-sensitized solar cells (QDSSCs) have attracted worldwide scientific and technological interest [1]. Basically, the structure of a QDSSC includes photoanode (a layer of porous oxide semiconductor with wide bandgap covered by semiconductor QDs as sensitizers), liquid electrolyte, and counter electrode. Many factors such as morphologies of oxide semiconductors, selection of sensitizers, and counter electrodes et al. could greatly affect the photoelectric conversion efficiency (PCE) of QDSSCs. Therefore, many efforts have been devoted to investigate these factors. Recently, a PCE of 9.01 % was achieved using CdSe_0.65_Te_0.35_ quantum dot (QD) as sensitizers [2]. However, the PCE of QDSSC is still far behind its theoretical efficiency, and further researches from different aspects are still required to improve the efficiencies of QDSSCs.

TiO_2_ is one of the most important semiconductors as the photoanode material which is the key components in the configuration of QDSSCs. Since the breakthrough work on colloidal TiO_2_ based DSSCs by O’Regan and Grätzel in 1991, various TiO_2_ nanostructures have been used in QDSSC, like nanoparticles, nanosheet, and nanorod [3–8]. Among them, single-crystalline TiO_2_ nanorod array would be one of the most desirable nanostructures for preparing photoanode of QDSSC due to its effective charge transfer property as well as excellent light harvesting ability. Inorganic semiconductors QDs such as CdS, PbS, PbSe, CdTe, CdSe, and Bi_2_S_3_ have been used to assist as a sensitizer for solar devices [9]. Among them, CdS is considered to be one of the potential photovoltaic semiconductive materials for its broadly tunable bandgap. The combination of wide bandgap semiconductors and CdS QDs can preferably collect the visible light used in photoelectrochemical applications.

One of obstacles which limit the performance of QDSSCs is the photogenerated carrier recombination. In order to restrain such recombination, introducing a passivation layer such as Al_2_O_3_ and ZnS between photoanode and electrolyte would be an effective method [10], which can retard the recombination by partially separating the electrons and electrolyte. Recently, graphitic carbon nitride (g-C_3_N_4_) has drawn much attention as a metal-free photocatalyst due to high photocatalytic efficiency.[11–13] Due to the band structure of g-C_3_N_4_, type II band alignment could be formed between g-C_3_N_4_ and TiO_2_, which can significantly prevent the migration of photogenerated electrons from TiO_2_ and QDs to the electrolyte [14]. Moreover, introducing g-C_3_N_4_ could expand the absorption range of sunlight. Therefore, introducing g-C_3_N_4_ into TiO_2_-based photoanodes should improve the performance of QDSSCs.

However, most reports about applications of g-C_3_N_4_ are for photocatalysts, and few works for solar cells could be found. Very recently, Wu et al. reported the improved short circuit current of ZnO-based dye-sensitized solar cells (DSSCs) using g-C_3_N_4_ as multifunctional protecting layer of ZnO particles [15]. Xu et al. reported enhanced PCE of DSSCs using g-C_3_N_4_ modified TiO_2_ nanosheets [16]. In present work, we investigated the effect of g-C_3_N_4_ as both recombination retarding layer and sensitizer on the performance of QDSSC. Single crystal TiO_2_ nanorod array was prepared by hydrothermal method on a transparent conducting glass and spin-coated with g-C_3_N_4_, leading to the formation of g-C_3_N_4_/TiO_2_ heterostructure. Compared with pure TiO_2_ nanorod array photoanodes, the g-C_3_N_4_ modified photoanodes showed an obvious improvement in cell performances. The results of I-V characteristic exhibited that introducing g-C_3_N_4_ increased both the open circuit voltage and short circuit photocurrent density, and the possible mechanism is discussed.

## Methods

### Materials

FTO glasses were purchased from Zhuhai Kaivo Optoelectronic Technology Co., Ltd. Acetone, ethanol, hydrochloric acid, and cadmium acetate and methanol were purchased from Beijing Chemical Works. Titanium butoxide was purchased from Shanghai Chemicals. Melamine, Na_2_S, S, urea, and acetic acid were purchased from Aladdin. CuSO_4_ was acquired from Tianjin Guangfu Technology Development Co., Ltd. Na_2_S_2_O_3_ was purchased from Damao Chemical Reagent, Tianjin.

### Preparation of TiO_2_ Nanorod Arrays

TiO_2_ nanorod array was fabricated according to the previous report [17]. Typically, 15 mL of deionized water was mixed with 15 mL hydrochloric acid. The mixture was then stirred for 15 min followed by the addition of 0.5 mL of titanium butoxide. The mixture was transferred into a 45-mL autoclave. Then, cleaned FTO substrates were put into the autoclave, and the hydrothermal process was conducted at 150 °C for 12 h.

### Preparation of g-C_3_N_4_ Paste

The g-C_3_N_4_ was prepared using the method reported previously [18–20]. Briefly, 3 g melamine and 4 g urea were mixed in a 20-mL crucible, transferred into a muffle furnace, and heated to and kept at 550 °C for 2 h. The yellow crystalline g-C_3_N_4_ bulk was obtained and then fully grinded into pale yellow powders. The g-C_3_N_4_ paste was prepared by mixing g-C_3_N_4_ powders (0.8 g), ethyl cellulose (0.4 g), and α-terpinol (3.245 g) in anhydrous ethanol (8.5 mL) and stirring the mixture for 24 h.

### Preparation of CdS/g-C_3_N_4_/TiO_2_ Photoanodes

**Scheme 1:**
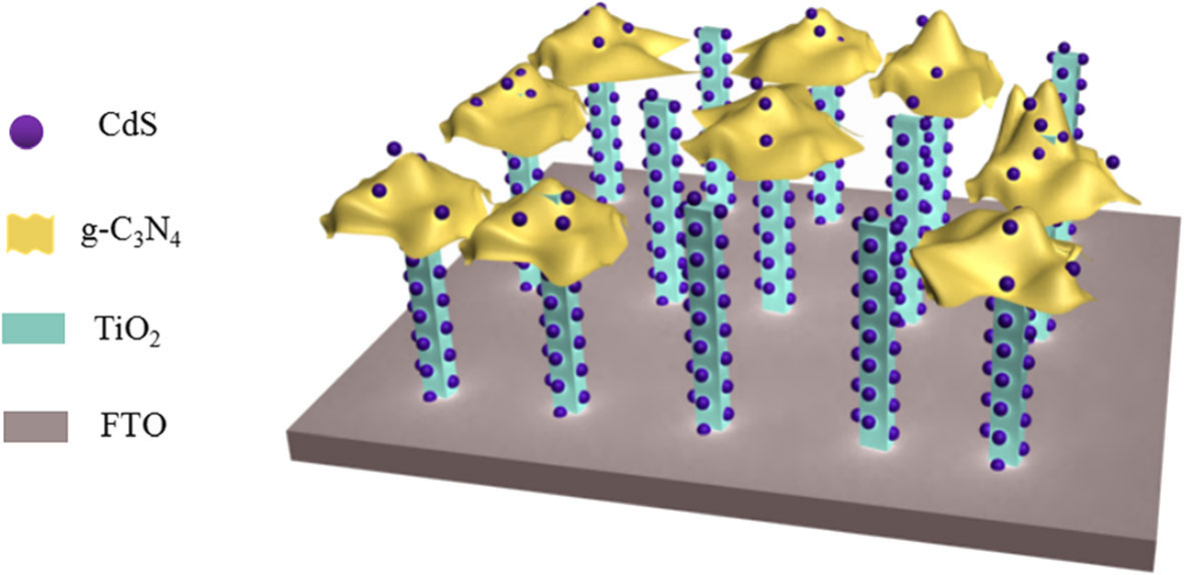
Schematic illustration of CdS/g-C_3_N_4_/TiO_2_ nanorod photoanode structure

The g-C_3_N_4_ paste was spin-coated on the as-prepared TiO_2_ nanorod. The as-received g-C_3_N_4_/TiO_2_ nanorod photoanodes were subjected to a sintering process in air at 450 °C for 30 min. After cooling to room temperature, the photoanodes were decorated with CdS QDs by successive ionic layer adsorption and reaction (SILAR) method [21]. The g-C_3_N_4_/TiO_2_ nanorod photoanode was successively dipped in a 0.05 M cadmium acetate methanol solution and a 0.05 M Na_2_S methanol solution each for 30 s. The two-step dipping procedure was termed as one cycle. The illustration of photoanode structure is shown in Scheme 1.

### Preparation of CuS Counter Electrodes

CuS counter electrodes were made by chemical bath deposition (CBD) method. One molar CuSO_4_ aqueous solution and 1 M Na_2_S_2_O_3_ aqueous solution were mixed with the volume ratio of 1:4. The pH of the mixed solution was adjusted to 2 with acetic acid. Then, the FTO glasses were immersed into 100 mL as-prepared mixed solution. The above solution was heated to 70 °C and kept for 4 h. After cooling down to the room temperature, the substrates were washed and dried in air and then heated to 130 °C and kept for 30 min.

### Fabrication of QDSSCs

The as-prepared CdS/g-C_3_N_4_/TiO_2_ nanorod photoanode and CuS counter electrode were assembled to a sandwich-type cell and penetrated with a polysulde electrolyte that consisted of 1 M Na_2_S and 1 M S in methanol and H_2_O solution (*v*/*v* = 7:3).

### Characterization

The samples were characterized using field emission scanning electron microscopy (FESEM, S4800, Hitachi), transmission electron microscopy (TEM) (Tecnai F20), X-ray diffraction (XRD) (D-MAX II A X-ray diffractmeter), X-ray photoelectron spectra (XPS) (VG ESCALAB MKII), and Fourier transform infrared spectroscopy (FTIR) (VERTEX 70). The cell performances were investigated by AM 1.5 solar simulator and Solar Cell Scan 100 (Zolix, Beijing).

## Results and Discussion

**Fig. 1:**
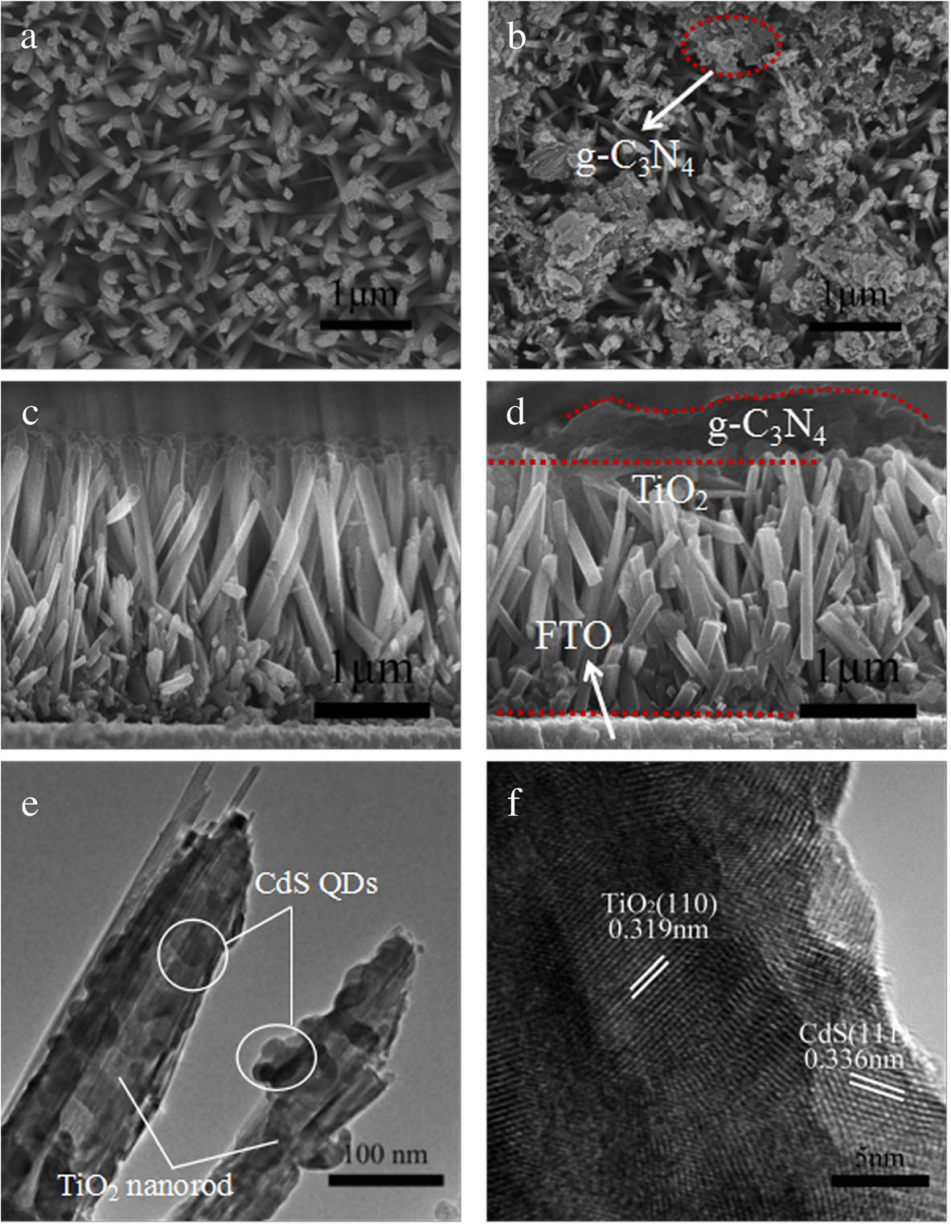
Morphologies of TiO_2_/FTO and g-C_3_N_4_/TiO_2_/FTO photoelectrodes: typical top view SEM images of TiO_2_/FTO photoelectrode (**a**) and g-C_3_N_4_/TiO_2_/FTO photoelectrode (**b**); typical cross-sectional view of the well-aligned TiO_2_ nanorod array (**c**) and g-C_3_N_4_/TiO_2_ photoelectrode (**d**); typical TEM image of single TiO_2_ nanorod deposited with CdS QDs (10 cycles) (**e**), and HRTEM of CdS QD decorated TiO_2_ nanorod (**f**)

The morphologies of the as-prepared TiO_2_ nanorods and g-C_3_N_4_/TiO_2_ nanorods on FTO substrate are shown in Fig. 1. As shown in Fig. 1a, TiO_2_ nanorods with high density in the average diameter ~100 nm are formed uniformly on FTO substrate. For these nanorods, while the side facets are smooth, the shape of top facets is square and composed of many step edges. These steps are responsible for further growth of the TiO_2_ nanorod, and these results show the expected growth habit of the tetragonal crystal. From the cross-sectional image of the sample as shown in Fig. 1c, it is obvious that the well-aligned nanorods are nearly normal to the FTO substrate. The length of the nanorods is about 3 μm. For TiO_2_ nanorods capped by g-C_3_N_4_, Fig. 1b shows that discontinuous g-C_3_N_4_ layer was coated on the surface of TiO_2_ nanorods. These remaining vacancies could assure that CdS quantum dots can be deposited on g-C_3_N_4_ as well as TiO_2_ nanorods. The cross-sectional view in Fig. 1d indicates that g-C_3_N_4_ was successfully coated on the TiO_2_ nanorods with the thickness of about 0.8 μm. Figure 1e shows the TEM image of TiO_2_ nanorod decorated with CdS QDs for 10 cycles. Compared with bare TiO_2_ nanorod, the rough surface could be observed after CdS QD deposition, indicating that large amounts of CdS QDs had been deposited on the TiO_2_ nanorods. This is further confirmed by HRTEM image (Fig. 1f). The lattice fringe space of 0.319 and 0.336 nm corresponds to the (110) plane of tetragonal rutile TiO_2_, and (111) planes of the cubic phase of CdS could be confirmed.

**Fig. 2:**
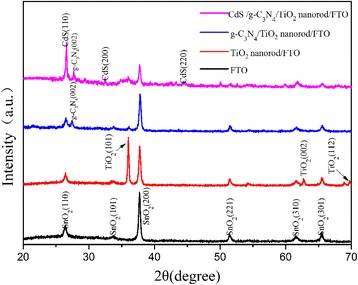
The XRD patterns for FTO substrate, TiO_2_/FTO, g-C_3_N_4_/TiO_2_/FTO, and CdS/g-C_3_N_4_/TiO_2_/FTO

**Fig. 3:**
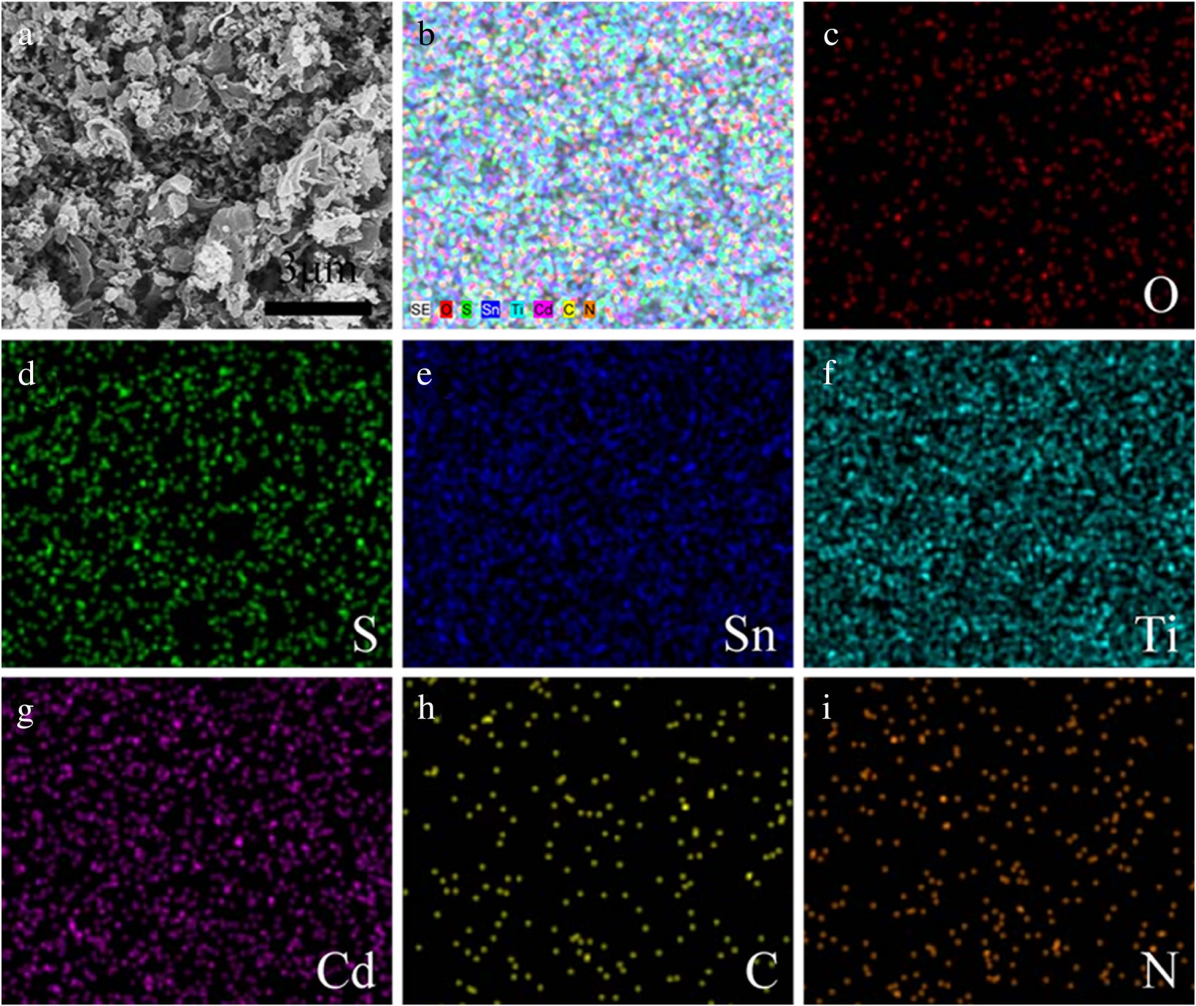
The EDX mapping images of CdS/g-C_3_N_4_/TiO_2_photoanode:SEM of CdS/g-C_3_N_4_/TiO_2_(a);EDX mapping of O,S,Sn,Ti,Cd,C,N(b);Element mapping of O(c),S(d),Sn(e),Ti(f),Cd(g),C(h),N(i)

Figure 2 shows the XRD curves of FTO substrate, TiO_2_/FTO, g-C_3_N_4_/TiO_2_/FTO, and CdS/g-C_3_N_4_/TiO_2_/FTO, respectively. The XRD result of TiO_2_/FTO exhibits a greatly increased (002) and (101) diffraction, suggesting the vertical growth of highly oriented titania nanorods on FTO, which is consistent with SEM observation. After coating with g-C_3_N_4_, a peak at 27.7° could be observed which is attributed to the typical (002) plane of the g-C_3_N_4_. After the deposition of CdS QDs, the XRD pattern of CdS/g-C_3_N_4_/TiO_2_/FTO shows diffraction peaks corresponding to the hexagonal wurtzite phase of CdS. Figure 3 shows the EDX mapping images of CdS/g-C_3_N_4_/TiO_2_ photoanode. The Sn comes from FTO substrate, and O is originated from FTO substrate and TiO_2_ nanorods. The same position of S and Cd indicates the CdS QD formation. The position distribution of C and N is similar, indicating the formation of g-C_3_N_4_ after spin coating. The EDX results are further confirmed by XPS.

**Fig. 4:**
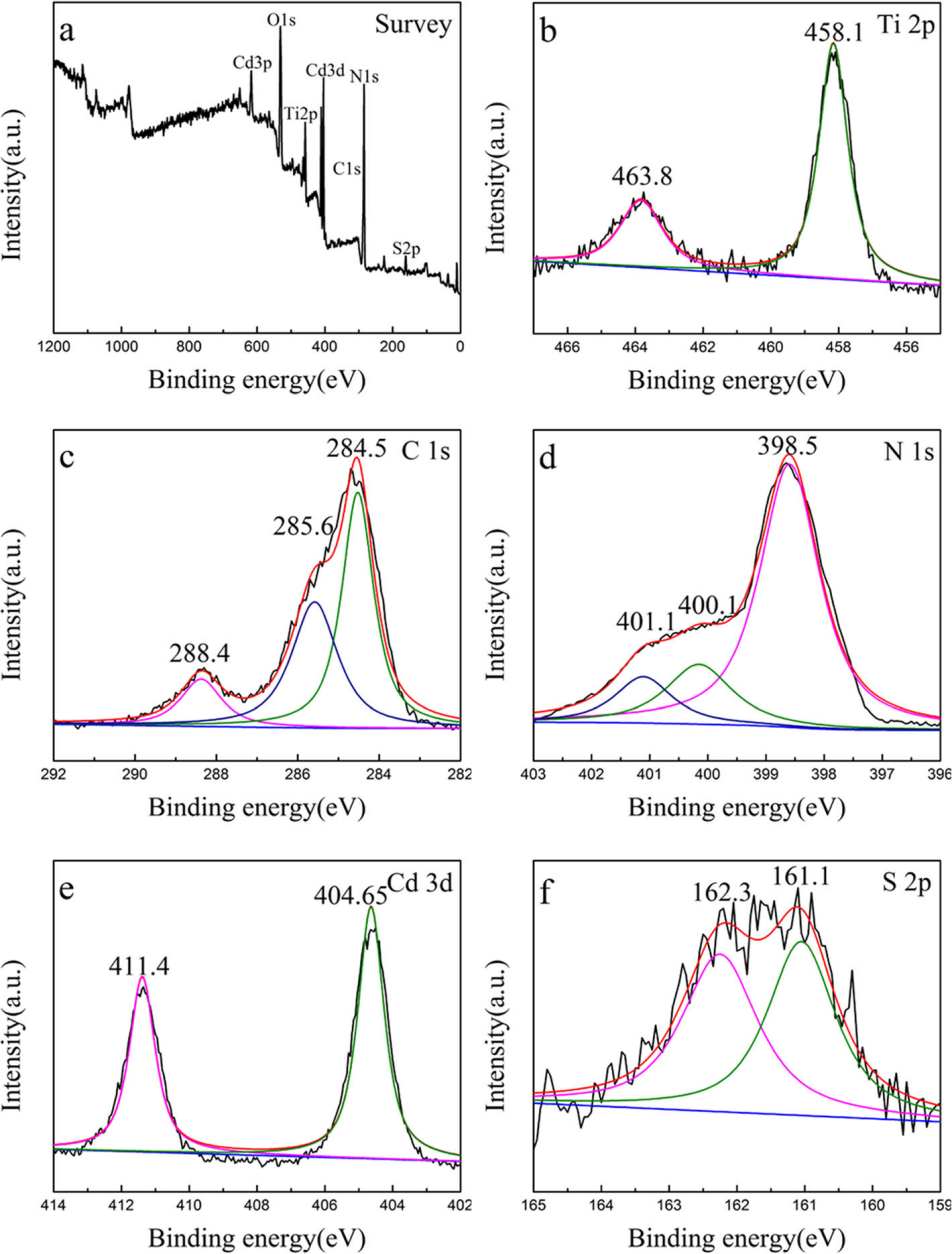
XPS spectrum of the as-prepared photoanode **a** survey, **b** Ti1s, **c** C1s, **d** N1s, **e** Cd 3d, and **f** S 2p

The XPS survey in Fig. 4a exhibits that the existence of C, N, Cd, S, Ti, and O in the CdS/g-C_3_N_4_/TiO_2_ photoanode. The Ti 2p3/2 and 2p1/2 centered at 458.1 and 463.8 eV are in agreement with those of pure TiO_2_ (Fig. 4b) [22–25]. The C 1s shown in Fig. 3c has three peaks situated at 284.5, 288.4, and 285.6 eV, which corresponds to sp2 C–C bonds, sp2-bonded carbon in N–C=N, and sp3-bonded carbon species, respectively. For N, three peak signals of N1s located at 398.5, 400.1, and 401.1 eV are present and attributed to sp2 bond N in triazine rings, tertiary N in N-(C)3 units, respectively [26]. These results indicate the presence of graphite-like C_3_N_4_. Moreover, the Cd 3d-related peaks at 404.65 and 411.4 eV are observed and attributed to Cd 3d5/2 and Cd 3d3/2, respectively. The S2p XPS spectra can be separated to two peaks at 161.1 and 162.3 eV which are ascribed to S^2−^ in CdS [27].

**Fig. 5:**
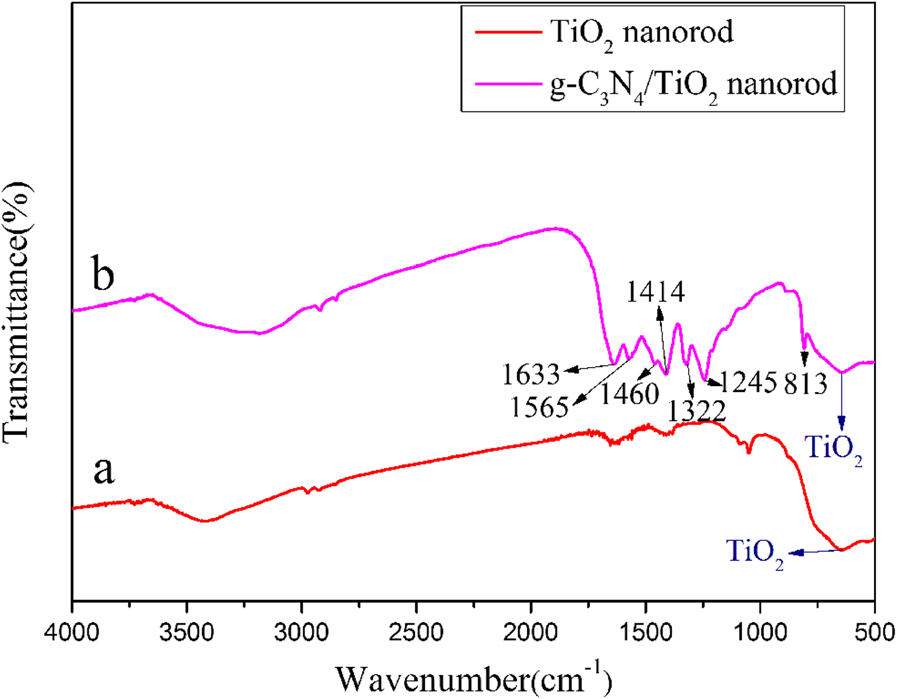
FTIR spectrum of bare TiO_2_ nanorod and g-C_3_N_4_/TiO_2_ nanorod

Figure 5 shows the comparison of the FTIR spectra of pure TiO_2_ nanorod and TiO_2_ nanorod/g-C_3_N_4_. The strong absorption between 500 to 800 cm^−1^ represents the bonds of Ti–O–Ti in both of the curves [28]. When g-C_3_N_4_ sheets are coated on TiO_2_ nanorods, several strong bands could be observed in the range of 1200–1700 cm^−1^ which are typical stretching modes of CN heterocycles [29]. Moreover, the peak at 813 cm^−1^ is due to variation of triazine units [30]. These absorption peaks once again confirm the existence of C_3_N_4_ on the as-prepared TiO_2_ nanorod photoanode.

**Fig. 6:**
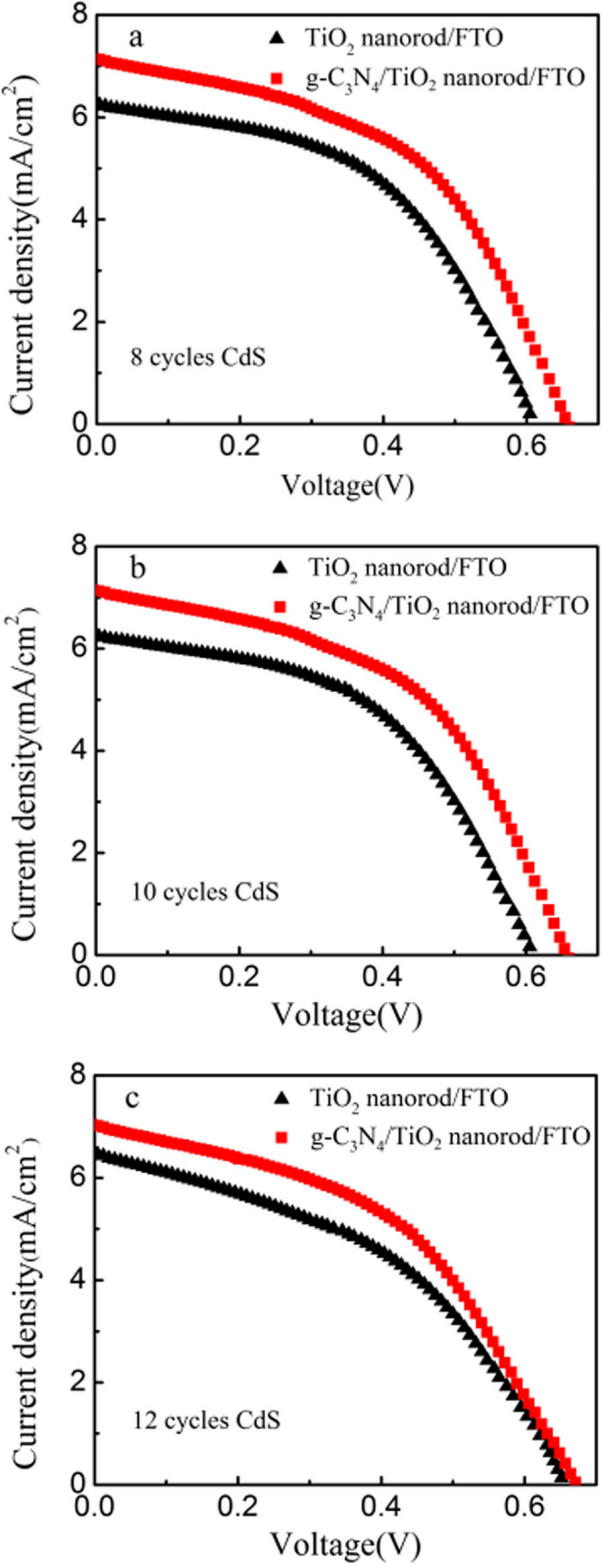
I-V curves acquired using CdS/TiO_2_ and CdS/g-C_3_N_4_/TiO_2_ photoanodes with different CdS QD SILAR cycles: 8 cycles (**a**), 10 cycles (**b**), and 12 cycles (**c**)

**Fig. 7:**
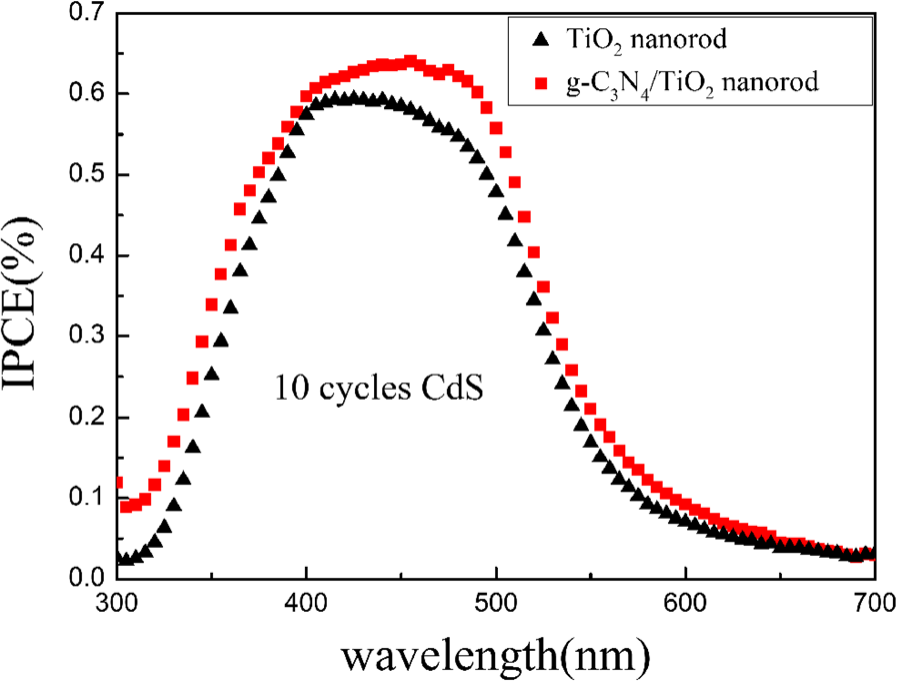
IPCE of 10 cycles CdS/TiO_2_ and 10 cycles CdS/g-C_3_N_4_/TiO_2_ electrodes

The cell performances are investigated as shown Fig. 6, and corresponding parameters are listed in Table 1. For both CdS/TiO_2_ and CdS/g-C_3_N_4_/TiO_2_ electrodes, the cell performances with different deposition cycles of CdS QDs are investigated. Both two kinds of electrodes exhibit the best performance with 10 cycles of CdS QD deposition, and the efficiency decreases with further increasing deposition cycles. This is probably due to the excessive deposition of QDs. If the deposition cycles of CdS QDs are more than 10, CdS QD with larger average size would be produced, and the aggregation and convergence among CdS QDs could happen at the surface of g-C_3_N_4_/TiO_2_. The larger CdS QDs would have poor ability to generate multiple excitons, originating from the disappearance of the quantum effect [31]. As shown in Table 1, the measurements of I-V characteristic indicate that the addition of g-C_3_N_4_ increases both the open circuit voltage and short circuit photocurrent density. As shown in Fig. 7, the photon-to-current conversion efficiency (IPCE) value is improved after coating g-C_3_N_4_ in the range of 300–600 nm. Compared with CdS/TiO_2_ electrode, it is worth noticing that the IPCE of CdS/g-C_3_N_4_/TiO_2_ electrodes is enhanced obviously between 400 and 500 nm. The maximum IPCE value occurs at ~470 nm which is very close to the bandgap of g-C_3_N_4_ used in this work. The improvement of IPCE could be due to the synergistic effect of g-C_3_N_4_ and CdS QD for sensitizing TiO_2_ nanorods.

**Table 1:** Photovoltaic performance parameters of CdS/TiO_2_ and CdS/g-C_3_N_4_/TiO_2_ electrodes

Samples	Cycles	Jsc (mA/cm^2^)	Voc (V)	FF	*η* (%)
TiO_2_ nanorod	8	5.86	0.56	0.51	1.67
	10	6.25	0.61	0.50	1.88
	12	6.48	0.65	0.43	1.83
TiO_2_ nanorod/g-C_3_N_4_	8	6.85	0.65	0.47	2.11
	10	7.13	0.66	0.49	2.31
	12	7.03	0.67	0.47	2.21

**Fig. 8:**
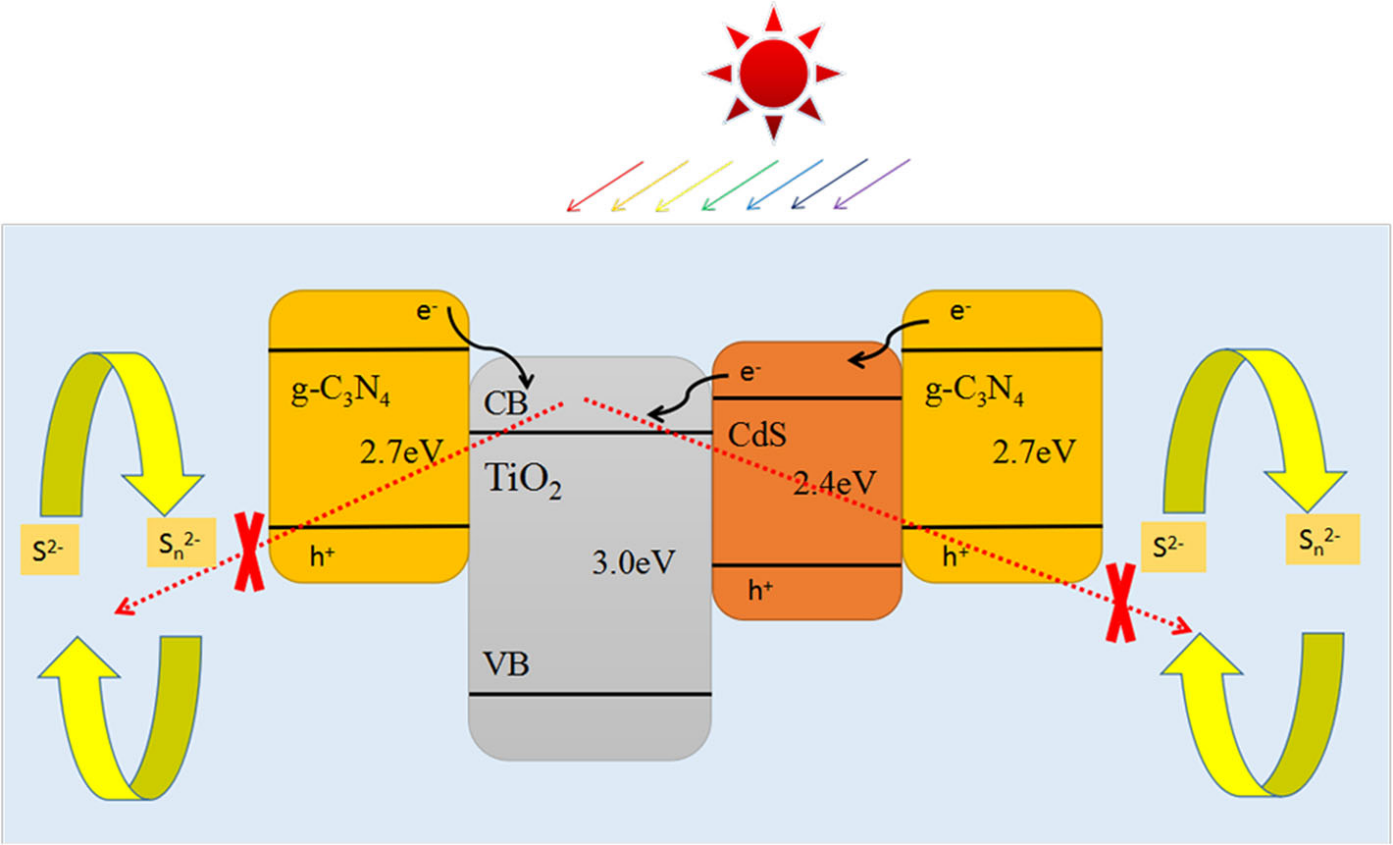
Mechanism of charge separation and transfer between g-C_3_N_4_ and TiO_2_ nanorod arrays under visible light irradiation

The mechanism of the performance improvement of QDSSCs in this work is suggested as below. As illustrated in Fig. 8, a type II band alignment between TiO_2_ and g-C_3_N_4_ could be built due to suitable band structure of g-C_3_N_4_. Therefore, the immigration of photogenerated electrons from the conduction band (CB) of TiO_2_ and CdS QDs to g-C_3_N_4_ and electrolyte would be restrained. The g-C_3_N_4_ layer on TiO_2_ nanorods acted as both block layer and effective light absorption layer could effectively promote the electron transport by retarding the backward recombination of electrons from TiO_2_ and electrolyte and also contribute additional electrons to increase the electron concentration in the photoanodes, thus to enhance the performance of QDSSCs. Moreover, the synergistic effect of g-C_3_N_4_ and CdS QDs for sensitizing TiO_2_ nanorods would be the other reason. As shown in IPCE measurement, introducing g-C_3_N_4_ will further improve photoelectron injection to TiO_2_ particularly in the range of 400–500 nm, which suggests that the existence of g-C_3_N_4_ layer will supplement the adsorption of sunlight. The matching conduction bands and valence bands of g-C_3_N_4_ and TiO_2_ greatly enhanced the separation and transfer of the photogenerated electrons and holes in the composite; thus, the photoelectrochemical performance of the g-C_3_N_4_/TiO_2_ electrode is improved.

## Conclusions

In summary, we introduce two-dimensional g-C_3_N_4_ layer in the single crystal TiO_2_ nanorod array photoanode. Compared with pure TiO_2_ nanorod array photoanodes, the g-C_3_N_4_ modified photoanodes showed an obvious improvement in cell performances, and a champion efficiency of 2.31 % was achieved, giving 23 % enhancement in cell efficiency. The improved performances were due to the matching conduction bands and valence bands of g-C_3_N_4_ and TiO_2_, which greatly enhanced the separation and transfer of the photogenerated electrons and holes and effectively suppressed interfacial recombination. Present work provides a new direction for improving the performance of QDSSCs.

## Abbreviations

CBD: Chemical bath deposition
DSSCs: Dye-sensitized solar cells
FESEM: Field emission scanning electron microscopy
FF: Fill factor
g-C_3_N_4_: Graphite carbon nitride
QDs: Quantum dots
QDSSCs: Quantum dot-sensitized solar cells
SILAR: Successive ionic layer adsorption and reaction
TEM: Transmission electron microscopy
XPS: X-ray photoelectron spectra

## References

[1] Chuang CM, Brown PR, Bulovic V, Bawendi MG (2014). Improved performance and stability in quantum dot solar cells through band alignment engineering. Nat Mater.

[2] Ren Z, Wang J, Pan Z, Zhao K, Zhang H, Li Y (2015). Amorphous TiO_2_ buffer layer boosts efficiency of quantum dot sensitized solar cells to over 9%. Chem Mater.

[3] Weng ZY, Guo H, Liu XM, Wu SL, Yeung KW, Chu PK (2013). Nanostructured TiO_2_ for energy conversion and storage. RSC Adv.

[4] Chen X, Jia B, Zhang Y, Gu M (2013). Exceeding the limit of plasmonic light trapping in textured screen-printed solar cells using Al nanoparticles and wrinkle-like graphene sheets. Light-Sci Appl.

[5] Jiu JT, Isoda S, Wang FM, Adachi M (2006). Dye-sensitized solar cells based on a single-crystalline TiO_2_ nanorod film. J Phys Chem B.

[6] Song MY, Ahn YR, Jo SM, Kim DY, Ahn J (2005). TiO_2_ single-crystalline nanorod electrode for quasi-solid-state dye-sensitized solar cells. Appl Phys Lett.

[7] Zhang XY, Sun SH, Sun XJ, Zhao YR, Chen L, Yang Y (2016). Plasma-induced, nitrogen-doped graphene-based aerogels for high-performance supercapacitors. Light-Sci Appl.

[8] Wang H, Bai YS, Zhang H, Zhang ZH, Li JH, Guo L (2010). CdS quantum dots-sensitized TiO_2_ nanorod array on transparent conductive glass photoelectrodes. J Phys Chem C.

[9] Bhande SS, Ambade RB, Shinde DV, Ambade SB, Patil SA, Naushad M (2015). Improved photoelectrochemical cell performance of tin oxide with functionalized-multiwalled carbon nanotubes-cadmium selenide sensitizer. ACS Appl Mater Interface.

[10] Lee YL, Lo YS (2015). Highly efficient quantum-dot-sensitized solar cell based on co-sensitization of CdS/CdSe. Adv Funct Mater.

[11] Ayan-Varela M, Villar-Rodil S, Paredes JI, Munuera JM, Pagán A, Lozano-Pérez AA (2015). Investigating the dispersion behavior in solvents, biocompatibility, and use as support for highly efficient metal catalysts of exfoliated graphitic carbon nitride. ACS Appl Mater Interface.

[12] Wang XC, Maeda K, Thomas A, Takanabe K, Xin G, Carlsson JM (2009). A metal-free polymeric photocatalyst for hydrogen production from water under visible light. Nat Mater.

[13] Chen HM, Xie YH, Sun XQ, Lv ML, Wu FF, Zhang L (2015). Efficient charge separation based on type-II g-C_3_N_4_/TiO_2_-B nanowire/tubes heterostructure photocatalysts. Dalton Trans.

[14] Low JX, Cao SW, Yu JF, Wageh S (2014). Two-dimensional layered composite photocatalysts. Chem Commun.

[15] Wu DP, Cao K, Wang FJ, Wang HJ, Gao ZY, Xu F (2015). Two dimensional graphitic-phase C_3_N_4_ as multifunctional protecting layer for enhanced short-circuit photocurrent in ZnO based dye-sensitized solar cells. Chem Eng J.

[16] Xu J, Wang GX, Fan JJ, Liu BS, Cao SW, Yu JG (2015). gC_3_N_4_ modified TiO_2_ nanosheets with enhanced photoelectric conversion efficiency in dye-sensitized solar cells. J Power Sources.

[17] Kim H, Lee J, Yantara N, Boix PP, Kulkarni SA, Mhaisalkar S (2013). High efficiency solid-state sensitized solar cell-based on submicrometer rutile TiO_2_ nanorod and CH_3_NH_3_PbI_3_ perovskite sensitizer. Nano Lett.

[18] LiaoYL ZSM, Ma J, Sun ZH, Yin C, Zhu CL (2014). Tailoring the morphology of g-C_3_N_4_ by self-assembly towards high photocatalytic performance. ChemCatChem.

[19] Ge L, Han CC (2012). Synthesis of MWNTs/gC_3_N_4_ composite photocatalysts with efficient visible light photocatalytic hydrogen evolution activity. Appl Catal B.

[20] Dai K, Lu LH, Liang CH, Liu Q, Zhu GP (2014). Heterojunction of facet coupled gC_3_N_4_/surface-fluorinated TiO_2_ nanosheets for organic pollutants degradation under visible LED light irradiation. Appl Catal B.

[21] Baker DR, Kamat PV (2009). Photosensitization of TiO_2_ nanostructures with CdS quantum dots: particulate versus tubular support architectures. Adv Funct Mater.

[22] Chang F, Zhang J, Xie YC, Chen J, Li CL, Wang J (2014). Fabrication, characterization, and photocatalytic performance of exfoliated gC_3_N_4−_TiO_2_ hybrids. Appl Surf Sci.

[23] Wang JX, Huang J, Xie HL, Qu AL (2014). Synthesis of gC_3_N_4_/TiO_2_ with enhanced photocatalytic activity for H_2_ evolution by a simple method. Int J Hydrogen Energy.

[24] Yan SC, Li ZS, Zou ZG (2009). Photodegradation performance of g-C_3_N_4_ fabricated by directly heating melamine. Langmuir.

[25] Samanta S, Martha S, Parida K (2014). Facile synthesis of Au/g-C_3_N_4_ nanocomposites: an inorganic/organic hybrid plasmonic photocatalyst with enhanced hydrogen gas evolution under visible-light irradiation. ChemCatChem.

[26] Li YG, Wei XL, Li HJ, Wang RR, Feng J, Yun H, Zhou AN (2015). Fabrication of inorganic–organic core-shell heterostructure: novel CdS@gC_3_N_4_ nanorod arrays for photoelectrochemical hydrogen evolution. RSC Adv.

[27] Bai J, Li JH, Liu YB, Zhou BX, Cai WM (2010). A new glass substrate photoelectrocatalytic electrode for efficient visible-light hydrogen production: CdS sensitized TiO_2_ nanotube arrays. Appl Catal B.

[28] Mamakhel A, Tyrsted C, Bojesen ED, Hald P, Iversen BB (2013). Direct formation of crystalline phase pure rutile TiO_2_ nanostructures by a facile hydrothermal method. Cryst Growth Des.

[29] Bojdys MJ, Müller JQ, Antonietti M, Thomas A (2008). Ionothermal synthesis of crystalline, condensed, graphitic carbon nitride. Chem Eur J.

[30] Tan GQ, Li ZP, Yuan HY, Dan X (2014). Sorption of cadmium from aqueous solution with a highly effective sorbent-B-doped g-C_3_N_4_. Sep Sci Technol.

[31] Su FL, Lu JW, Tian Y, Ma XB, Gong JL (2013). Branched TiO_2_ nanoarrays sensitized with CdS quantum dots for highly efficient photoelectrochemical water splitting. Phys Chem Chem Phys.

